# Longitudinal Assessment of Physical Activity, Fitness, Body Composition, Immunological Biomarkers, and Psychological Parameters During the First Year After Diagnosis in Women With Non-Metastatic Breast Cancer: The BEGYN Study Protocol

**DOI:** 10.3389/fonc.2021.762709

**Published:** 2021-10-19

**Authors:** Cosima Zemlin, Caroline Stuhlert, Julia Theresa Schleicher, Carolin Wörmann, Laura Altmayer, Marina Lang, Laura-Sophie Scherer, Ida Clara Thul, Carolin Müller, Elisabeth Kaiser, Regine Stutz, Sybelle Goedicke-Fritz, Laura Ketter, Michael Zemlin, Gudrun Wagenpfeil, Georges Steffgen, Erich-Franz Solomayer

**Affiliations:** ^1^ Department for Gynecology, Obstetrics and Reproductive Medicine, Saarland University Medical Center, Homburg, Germany; ^2^ Department for General Pediatrics, Saarland University Medical Center, Homburg, Germany; ^3^ Department of Behavioural and Cognitive Sciences, Institute for Health and Behaviour, University of Luxembourg, Esch-sur-Alzette, Luxembourg; ^4^ Institute for Medical Biometry, Epidemiology and Medical Informatics (IMBEI), Saarland University, Homburg, Germany

**Keywords:** breast cancer, physical activity, spiroergometry, psychological parameters, body composition, chemotherapy, immune monitoring, observational study

## Abstract

**Background:**

Moderate physical activity is associated with an improved prognosis and psychosocial outcome in breast cancer patients. Although exercise and physical activity are associated with multiple physiological and psychological effects, many of the underlying mechanisms remain obscure. The BEGYN study (Influence of physical activity in breast cancer patients on physiological and psychological parameters and on biomarkers) aims at identifying potential associations between the extent of physical activity, fitness, body composition, immunological biomarkers, psycho-emotional parameters, and the course of treatment during the first year after diagnosis of breast cancer.

**Methods:**

The prospective observational BEGYN study will include 110 non-metastatic breast cancer patients. The patients will be assessed during a base line visit prior to the initiation of the antineoplastic therapy and after 3, 6, 9 and 12 months. The physical activity will be measured using a fitness tracker and a self-assessment diary during the entire study. Each visit will include the assessment of (i) cardiorespiratory fitness measured by spiroergometry, (ii) body composition, (iii) psycho-emotional parameters (quality of life, mental health, fatigue, depression, distress, anxiety, well-being), and (iv) extensive blood tests including routine laboratory, vitamin D, selenium and immunologically relevant biomarkers (e.g., leukocyte subpopulations and cytokine profiles).

**Discussion:**

Whereas most studies investigating the influence of physical activity in breast cancer patients focus on specific activities for three months or less, the BEGYN study will quantify the daily physical activity and cardiorespiratory fitness of breast cancer patients based on objective measurements in the context of the oncological therapy for 12 months after diagnosis. The study will reveal potential associations between exercise, immune status and physical as well as psycho-emotional outcome and the clinical course of the disease. Moreover, complementary therapies such as Vit D and Selenium supplementation and parameters investigating the motivation of the patients are part of the study. Due to this holistic approach, the BEGYN study will guide towards confirmatory studies on the role of physical activity in breast cancer patients to develop individualized counselling regarding the recommended type and extent of exercise.

**Trial Registration:**

This study has been registered at the German Clinical Trials Register DRKS00024829.

## 1 Introduction

Historic recommendations to avoid physical activity during cancer treatment to save all energy for fighting the disease have proven wrong (1–6). Breast cancer is the most common cancer in women, accounting for more than 680.000 deaths per year worldwide (7). Although modern breast cancer treatment such as improved diagnostic and staging procedures, advanced systemic therapy, surgery and radiotherapy increases long-term survival and clinical outcome of breast cancer patients, there is still a deficiency of supportive and psychosocial care (8). Breast cancer survivors are at risk of suffering potentially disabling physical and psychological sequelae, such as lymphedema, axillary web syndrome, chronic pain, osteoporosis and fractures, arthralgia, chronic fatigue syndrome and depression (9). During and after antineoplastic therapy, rehabilitation and complementary therapies are crucial to improve the quality of life and overall prognosis of breast cancer survivors (10–12).

Multiple studies in cancer patients have demonstrated that physical activity and exercise correlate with an improved outcome regarding the course of the underlying disease and with a better tolerance to the antineoplastic treatments (13). For example, exercise had positive effects on the fatigue syndrome and quality of life in cancer (12, 14), the course of lymphedema (13, 15) and osteoporosis in breast cancer (16) and prostate cancer (17). Moreover, physical activity influences various functions of the immune system, such as the proportions of circulating leukocyte subsets and the expression of cytokines (4, 18–21). Moderate sporting activity has an immune-protective effect, whereas excessive sporting activity is associated with an increased susceptibility to infections - possibly mediated by a reduction in circulating natural killer cells (22). Natural killer cells and other leukocyte subsets play a crucial role in the physiological attempts of the organism to control cancer cells (23). Thus, Ashcraft et al. put forth the hypothesis that exercise-induced modulations of the immune status do not only alter the susceptibility to infections, but also the immune response to neoplastic diseases and the effectivity of antineoplastic therapies (24). Current data suggest that a complex interplay of the above-mentioned factors contributes to a positive correlation between the quantity of physical activities and event-free survival in cancer patients (69% reduced hazard of mortality among highly active patients) (25). In consequence, more prospective studies were recommended to characterize the influence of sporting activities on the immune system and on potential individualized rehabilitation approaches in cancer patients (9, 26, 27).

According to Mehnert et al. (28) the prevalence of any mental disorder among the major tumor entities is 32% and breast cancer has the highest prevalence of mental disorders with 42%. 17% of breast cancer patients are afflicted with anxiety disorders and 9% with affective disorders. Physical activity can affect anxiety and depression in breast cancer patients. Interestingly, leisure time physical activity was negatively related to depression, whereas occupational physical activity related positively to anxiety (29).

30% of disease-free breast cancer survivors suffer from cancer related fatigue syndrome, causing a massive reduction of psycho-emotional wellbeing and quality of life (30–32). Regular exercise plays an important role in the management of cancer-related fatigue (33–35). Clinical trials have shown that individual and patient-adapted exercise programs yield the best outcome regarding physical functioning and health among breast cancer patients (25, 32, 36–38).

The body composition strongly correlates with physical activity and is a relevant prognostic factor for breast cancer patients (39). On average, overweight patients have a higher risk to develop breast cancer, a higher rate of relapses and a shorter recurrence-free period (40). The loss of muscle mass and the gain of fat mass (sarcopenia) can be detected with a bioelectrical impedance analysis, a simple, non-invasive technique (41). A lower muscle index can be associated with a higher toxicity of the chemotherapy (42).

Since the physical activity plays a key role in determining the prognosis of breast cancer patients, it is useful to combine exercise diaries with objective measurements such as pedometers or fitness trackers (43, 44). Moreover, this yields continuous information on vital parameters, including the pulse and resting heart rate as an estimate for overall cardiopulmonary fitness (45).

Studies that combine fitness tests, physical assessments, immunological markers, and psychological tests in breast cancer patients are still scarce, thus it is highly difficult to understand potential cross-links between these aspects. Filling this gap of knowledge could allow conclusions on individualized prevention and rehabilitation of the multiple sequelae that are potentially associated with breast cancer (9). Clinical studies in cancer patients must consider that according to current guidelines, all breast cancer patients are to be advised to exercise endurance and muscle strength (39). However, little is known on the extent to which patients adhere to this recommendation. Moreover, it would be unethical to withhold motivation for sportive activity from breast cancer patients for experimental purposes as a control. Thus, the BEGYN study was designed as an observational study, using validated methods to assess physical activity, cardiopulmonary fitness, body composition, psychological parameters, and extensive blood tests (e.g., immune status, vitamin D and selenium) during the first year of antineoplastic therapy after diagnosis of non-metastatic breast cancer. To gain information even after a longer period the BEGYN study is designed to collect data over one year after diagnosis. This study will shed light into the dynamics of physiological and psychological variables and the course of the disease during the first year after initiation of antineoplastic therapy in breast cancer patients in correlation with the physical activity. Ultimately, this study will lay the basis to develop individualized recommendations for exercise in breast cancer patients to improve the quality of life and prognosis.

## 2 Materials and Methods

### 2.1 Study Population

The BEGYN study will assess 110 female patients with non-metastatic invasive breast cancer prior to the initiation of antineoplastic therapy. The patients will be followed for one year after diagnosis. Since the study focuses on variables that are heavily influenced by gender (e.g., physical fitness and body composition), we did not include male breast cancer patients. Inclusion and exclusion criteria ensure that patients can undergo spiroergometry, blood tests, assessment of physical activities and psychological parameters during the first year after the diagnosis of breast cancer. The detailed inclusion criteria and exclusion criteria are given in **Table 1**.

**Table 1:** Inclusion criteria and exclusion criteria of the BEGYN study.

Inclusion criteria	Exclusion criteria
Female sex Age ≥ 18 years Invasive, non-metastatic breast cancer Sufficient language skills to fill the questionnaires and to write an activity diary Sufficient technical skills or support for using a smart phone and fitness tracker Given written informed consent	Any antineoplastic treatment or invasive procedures (e.g., surgery for venous port) prior to baseline measurement Life expectancy < 12 months Non-invasive disease (e.g., carcinoma *in situ*) Previous or current history of other neoplasia Inability to perform a spiroergometry on a tread mill Pregnant or nursing women

### 2.2 Study Schedule

**Figure 1:**
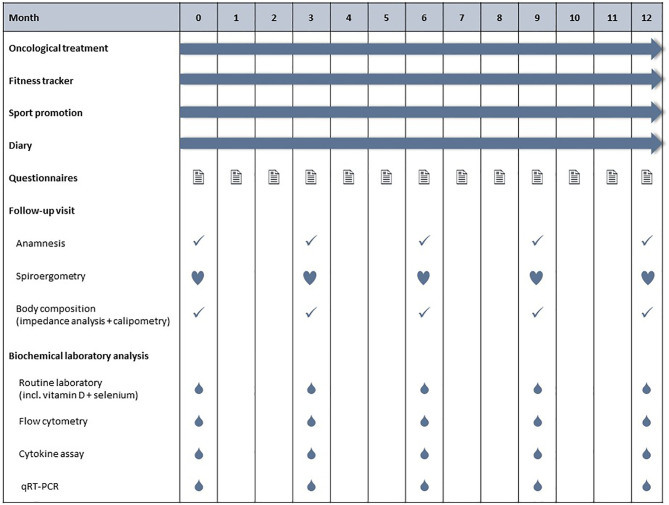
Schedule for the BEGYN study. All patients are asked to write down their physical activities in a diary (type and duration of exercise) and to continuously wear a fitness tracker. Study visits are scheduled quarterly, with the first visit taking place before initiation of antineoplastic therapy.

The patients are enrolled to the BEGYN study after initial diagnosis. The baseline study visit is scheduled before the initiation of any antineoplastic therapy, followed by quarterly follow up visits. During each visit, patients undergo a clinical assessment, spiroergometry on a treadmill, blood tests, measurement of the body composition by bioimpedance analysis, plicometry, validated psychological questionnaires and other assessments (**Figure 1**).

### 2.3 Clinical Assessment

Routine assessment during quarterly follow-up visits includes an anamnesis focused on potential side effects of antineoplastic therapies, measuring the body weight and blood pressure.

### 2.4 Assessment of Physical Activity and Exercise

#### 2.4.1 Patient Self-Assessment Diary

Each patient will be asked to document her daily sporting activities in a standardized self-assessment study diary. The patients will be instructed by study personnel how to use the diary during recruitment and during each follow-up visit. The study personnel check the completeness and accuracy of the diary and discuss potential improvements with the patients on each follow-up visit. The diary will be used to document the following points:

- Medically relevant information (change of medication or nutritional supplements, fever, symptoms etc.)
- Daily sportive activities
- Weekly read outs of the fitness tracker
- Weekly psychological questionnaires
- Questions and notes that the study participants might have.

As a standardized measure of physical activity the metabolic equivalent task (MET) will be used to describe the metabolic turnover of the patients even when performing different sportive activities (46, 47).

#### 2.4.2 Spiroergometry

During each of the five study visits the patients perform a spiroergometry on a treadmill (XRCISE RUNNER MED™ by Cardiowise, ERGO-FIT™, Pirmasens, Germany) for the assessment of cardiopulmonary fitness (48). After a technical introduction for the patient, the calibration of the aeroman™ and a baseline spirometry in seated position, the patients start walking at an individually determined speed, typically 4 kmph. Subsequently, the patient is challenged according to a standardized, validated protocol that ensures a linear increase in Oxygen uptake response (49). For inter- and intraindividual comparison, the time course of work rate in watts (WR_(t)_) is calculated using the formula published by Porszasz et al. (49) WT(t) = m * g * ν(t) * sin (α), where m is body mass in kg, g is the gravitational acceleration (9.81 m*s^-2^), ν(t) is the time course of velocity in meters per second, and α is the angle of inclination. The speed or gradient of the treadmill is increased by 0.5 kmph or 1 percent every 2 minutes to intensify the level of exertion. After each interval, the patient breathes through a tightly fitting spirometer mouthpiece (aeroman™ professional, ACEOS GmbH, Fürth, Germany) according to a validated protocol (50). The measurement will be terminated once the patient reaches a maximum heart rate defined as 220 – age (bpm) or a respiratory quotient >1. Furthermore, the subjective perception of exertion according to Borg >17 (51), dizziness, dyspnea, nausea or pain will lead to termination of the spiroergometry (52). The patient can interrupt the measurement due to subjective exhaustion at any time. This will reveal intraindividual longitudinal changes during the first year after the diagnosis of breast cancer. The ventilatory threshold (VT) will be used as a submaximal indicator of general cardiopulmonary fitness (53). VT represents the excessive increase of carbon dioxide output compared to oxygen uptake. In addition, heart rate (HR), oxygen uptake (VO_2_), carbon dioxide release (VCO_2_), respiratory quotient (RQ), breathing frequency (BF) and respiratory minute volume (VE) will be assessed to allow the analysis of endurance performance. The measurements obtained with the spiroergometry are given in **Table 2**.

**Table 2:** Spiroergometry.

Description	Abbreviation	Unit
Heart rate	HR	Beats per minute
Oxygen uptake	VO_2_	L/min
Carbon dioxide release (l/min)	VCO_2_	L/min
Respiratory quotient	RQ	VO_2_/VCO_2_
Breathing frequency	BF	Breaths per minute
Respiratory minute volume	VE	L/min

#### 2.4.3. Fitness Tracker

Physical activity is assessed daily by supplying each patient uses a commercial fitness tracker (Fitbit charge 3™ (Fitbit Inc., San Francisco) that will be linked to her smartphone (54). Study personnel assists the patients and – if required – their associate to install the smartphone app and to setup the measurements. The patients are requested to transcribe the measurements of their fitness tracker into their study diary weekly. Those values are shown in **Table 3**.

**Table 3:** Measurements with the fitness tracker.

Description	Abbreviation	Registration interval	Unit
**Core stats**		**Daily**	
Steps taken		Daily	Count
Resting heart rate	RHR	Daily	Beats per minute
Calories burned	Cal	Daily	Kcal
**Workout stats**		**Real-time**	
Elapsed time		Real-time	Minute
Distance covered		Real-time	Km
Calories burned		Real-time	Kcal
Average heart rate	øHR	Real-time	Beats per minute
Maximum heart rate	HRmax	Real-time	Beats per minute

### 2.5 Body Composition

#### 2.5.1. Bioelectrical Impedance Analysis

The body composition will be determined based on bioelectrical impedance analysis (BIA) (TANITA scale™, Tanita Europe BV, Stuttgart). The test person stands barefoot on a body scale and holds sensors in both hands. Using the impedance between the four measuring points, numerous data can be collected that provide information on the muscle, fat, bone and water content of the whole body and individual compartments. Bioimpedance analysis is routinely used in the context of nutritional advice and sports medicine, but it can also shed light into disease processes, such as hemodialysis patients (55) (**Table 4**). When accessible, the body composition will also be estimated by using routine CT scans of the study patients as previously published (56).

**Table 4:** Measurements by scale and bioimpedance analysis.

Item	Unit
Weight	kg
Total body fat	%
Total muscle mass	kg
Bone mass	kg
Body-Mass-Index	Kg/m²
Basal metabolic rate	kcal
Metabolic age	years
Total body water	%
Visceral fat	kg
Segmental muscle mass in arms, legs, and torso	kg
Segmental body fat in arms, legs, and torso	%

#### 2.5.2 Plicometry (Calipometry)

**Figure 2:**
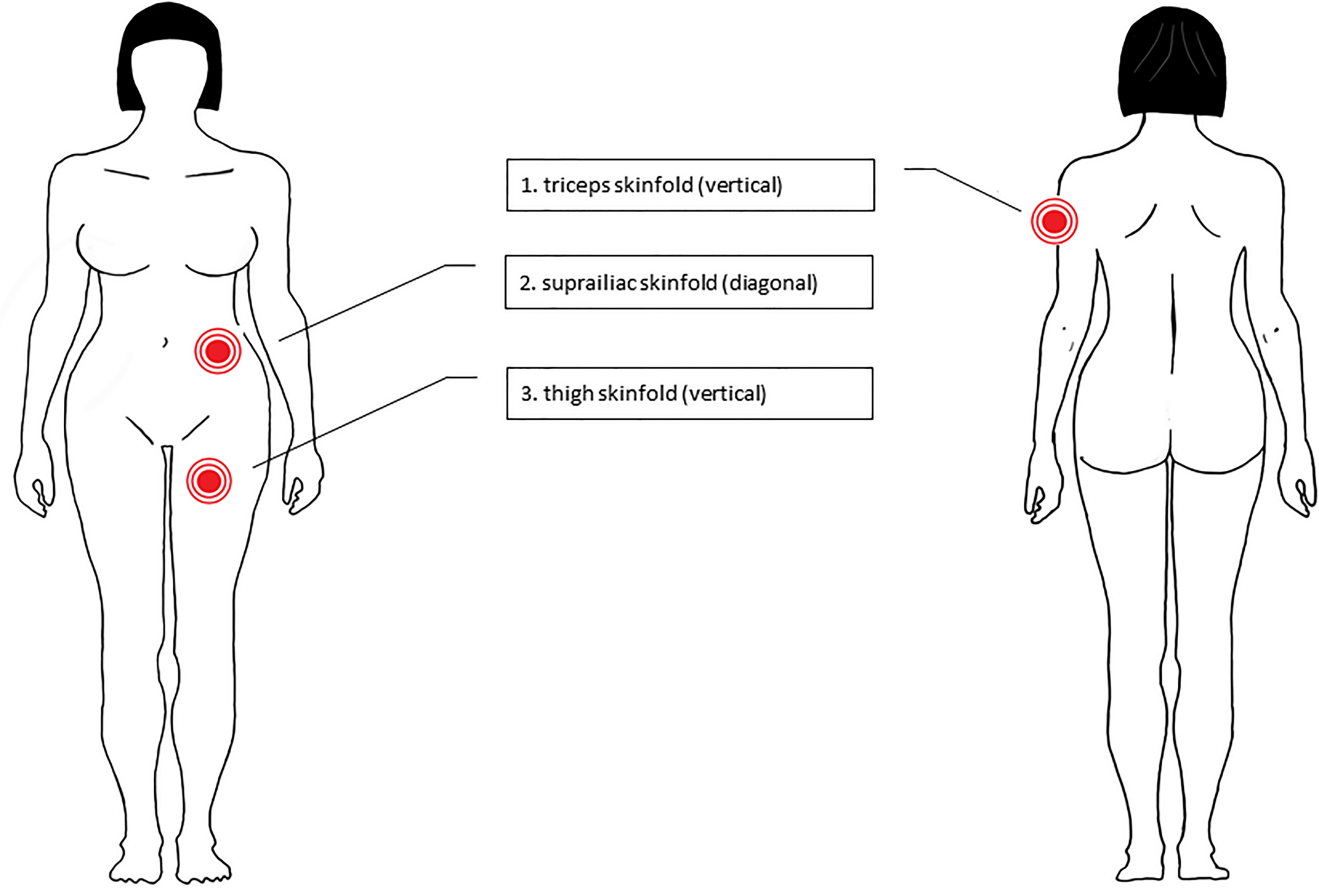
Plicometry sites of measurement (58, 59). Skinfold measurement using the 3 point-method (1 = triceps, 2 = suprailiac skinfold; 3 = thigh).

Plicometry allows a standardized estimate of the body fat status which can change under influences such as sport, chemotherapy or cancer (57–60). In the BEGYN study, fat distribution is assessed using the skinfold measurement using the 3 point-method (triceps, suprailiac skinfold, and thigh) according to Jackson and Pollock (61). (**Figure 2**). The total body subcutaneous fat tissue (in kg) was estimated from the plicometry measurements using the formula (61):


$$\text{Total}\;\text{body}\;\mathrm{subcutaneous}\;\text{fat}\;(\text{kg})\;=\;[(4.95/(1.0994921-(0.0009929^{*}\text{S})+(0.0000023^{*}{\text{S}}^{2})-(0.0001392^{*}\text{age}\{\text{in years}\})))-4.5{]}^{*}100.$$

S is the sum of the skinfold thicknesses measured at the triceps, the suprailiac skinfold and the thigh.

### 2.6 Nutritional Habits, Intake Of Food Supplements, Self-Medications, and Lifestyle

According to the guideline, a Mediterranean diet was recommended to all patients (39). Nutritional habits were assessed using a questionnaire with a focus on characteristic dietary patterns. In addition, sleeping, smoking, and drinking habits, the use of food supplements and self-medication as well as exposure to sunlight and use of sun protection were assessed using questionnaires and the self-assessment diary, respectively.

### 2.7 Biomarkers

Cancer, antineoplastic therapy as well as physical activity are closely related with biomarkers (22, 62–66).

#### 2.7.1 Blood Count, Biochemical Laboratory Markers

For disease monitoring and adjustment of the antineoplastic therapy, multiple biomarkers are routinely assessed during the quarterly study visits (**Table 5**). In addition, the BEGYN study will include vitamin D and selenium concentrations since they are often discussed as potentially relevant to cancer biology (69–72).

**Table 5:** Laboratory values, intervals of assessment and reference values.

Parameter	Assessment interval	Vial	Unit	reference value
**Blood count**				
Erythrocytes	quarterly	EDTA	10\S\12/l	4.00 – 5.20
Hb	quarterly	EDTA	g/dl	12.0 – 16.0
Leukocytes	quarterly	EDTA	10\S\9/l	3.9 – 10.2
Thrombocytes	quarterly	EDTA	10\S\9/l	140 – 400
**Differential leukocyte count**				
Neutrophils rel.	quarterly	EDTA	%	42.0 – 77.0
Neutrophils abs.	quarterly	EDTA	10\S\9/l	1.5 – 7.7
Lymphocytes	quarterly	EDTA	%	25.0 – 45.0
Monocytes	quarterly	EDTA	%	2.0 – 10.0
Eosinophils	quarterly	EDTA	%	0.0 – 5.0
Basophils	quarterly	EDTA	%	0.0 – 1.0
**Biochemical parameters**				
Sodium	quarterly	Li-Heparin-Plasma	mmol/l	135 – 145
Potassium	quarterly	Li-Heparin-Plasma	mmol/l	3.5 – 5.1
Calcium	quarterly	Li-Heparin-Plasma	mmol/l	2.2 – 2.6
Magnesium	quarterly	Li-Heparin-Plasma	mmol/l	0.66 – 1.07
Iron	quarterly	Li-Heparin-Plasma	µg/dl	33 – 193
Creatinin	quarterly	Li-Heparin-Plasma	mg/dl	0.50 – 0.90
Urea	quarterly	Li-Heparin-Plasma	mg/dl	17 – 48
Uric acid	quarterly	Li-Heparin-Plasma	mg/dl	2.5 – 5.7
Glucose	quarterly	Li-Heparin-Plasma	mg/dl	60 – 100
Protein	quarterly	Serum	g/l	66 – 87
Albumin	quarterly	Serum	g/l	35 – 52
Cholesterol	quarterly	Li-Heparin-Plasma	mg/dl	<200
Triglycerides	quarterly	Li-Heparin-Plasma	mg/dl	<150
CK	quarterly	Li-Heparin-Plasma	U/l	0 – 170
ASAT	quarterly	Li-Heparin-Plasma	U/l	0 – 35
ALAT	quarterly	Li-Heparin-Plasma	U/l	0 – 35
gamma-GT	quarterly	Li-Heparin-Plasma	U/l	<40
Alkaline phosphatase	quarterly	Li-Heparin-Plasma	U/l	35 – 104
Bilirubin	quarterly	Li-Heparin-Plasma	mg/dl	<1.2
Lipase	quarterly	Li-Heparin-Plasma	U/l	13 – 60
LDH	quarterly	Li-Heparin-Plasma	U/l	0 – 262
CRP	quarterly	Li-Heparin-Plasma	mg/l	0.0 – 5.0
HbA1c	quarterly	EDTA	%	<6.0
LDL-Cholesterol	quarterly	Li-Heparin-Plasma	mg/dl	<130
HDL-Cholesterol	quarterly	Li-Heparin-Plasma	mg/dl	45 – 65
VLDL-Cholesterol	quarterly	Li-Heparin-Plasma	mg/dl	<35
Interleukin-6	quarterly	Li-Heparin-Plasma	pg/ml	<7
Selenium	quarterly	Serum	µg/l	50 – 120 (67)
Vitamin D-25-OH	quarterly	Serum	ng/ml	30 – 100 (68)
**Hormones**				
TSH	quarterly	Li-Heparin-Plasma	µIU/ml	0.27 – 4,2
fT3	quarterly	Li-Heparin-Plasma	pg/ml	2.0 – 4.4
fT4	quarterly	Li-Heparin-Plasma	ng/dl	0.93 – 1.7
Cortisol	quarterly	Serum	µg/dl	4.82 – 19.5*
Insulin	quarterly	Serum	µIU/ml	<29.1
ß-hCG	quarterly	Serum	mIU/ml	<1.0
**Tumor marker**				
CA 15-3	optional	Serum	U/ml	<26.2

^*^Reference values for Cortisol vary depending on the time of blood collection. As in the BEGYN study, samples were routinely taken in the morning these standard values were used.

#### 2.7.2 Leukocyte Subsets

Peripheral blood mononuclear cells (PBMCs) will be obtained from 9,6 ml anticoagulated blood samples by Ficoll density gradient centrifugation and resuspended in 90% fetal calf serum + 10% dimethylsulphoxide. Plasma supernatant will be collected and all biosamples will be cryopreserved at -80°C until further processing (73).

The underlying disease, the antineoplastic therapies and physical activity can have a profound impact on the immune system, respectively (26, 74–77). The number of 36 circulating leukocyte subsets will be assessed by flow cytometry using a panel of validated antibody stainings (**Table 6**), based on previous studies (73, 78). Cells will be analyzed with FACSCelesta™ (BD Biosciences, Franklin Lakes, New Jersey, USA) (79).

**Table 6:** Leukocyte subsets.

population	Surface antigens
**B cells**	
innate B cells	CD19+ CD27- IgD- IgM-
naïve B cells	CD19+ CD27- IgD+ IgM+
memory B cells	CD19+ CD27+
marginal zone memory B cells	CD19+ CD27+ IgD+ IgM+
IgM memory B cells	CD19+ CD27+ IgD- IgM+
class switched memory B cells	CD19+ CD27+ IgD- IgM-
late memory B cells	CD19+ CD27+ CD38+ IgM+
plasmablasts	CD19+ CD27+ CD38^bright^ IgM-
transitonal B cells	CD19+ CD20+ CD27- CD38+
pre-naïve B cells (B1 cells)	CD20+ CD27+ CD43+ CD70-
B2 cells	CD20+ CD27+ CD43-
**T cells**	
Th cells with αβ-TCR	TCRαβ+ CD4+
cytotoxic Th cells with αβ-TCR	TCRαβ+ CD8+
memory effector Th cells	CD3+ CD4+ CD62L- CD45RO+
memory central Th cells	CD3+ CD4+ CD62L+ CD45RO+
naïve effector Th cells	CD3+ CD4+ CD62L- CD45RO-
naive central Th cells	CD3+ CD4+ CD62L+ CD45RO-
memory effector cytotoxic T cells	CD3+ CD8+ CD62L- CD45RO+
memory central cytotoxic T cells	CD3+ CD8+ CD62L+CD45RO+
naïve effector cytotoxic T cells	CD3+ CD8+ CD62L- CD45RO-
naïve central cytotoxic T cells	CD3+ CD8+ CD62L+ CD45RO-
T cells with γδ-TCR	TCR γδ+ CD3+ CD5+
naïve thymus negative Th cells	CD3+ CD4+ CD31- CD45RO-
naïve thymus negative Th cells	CD3+ CD4+ CD31+ CD45RO-
Th1 cells	CD3+ CD4+ CD183+ CCR6+
Th2 cells	CD3+ CD4+ CCR4+ CRTH2+
naïve Th1 cells	CD3+ CD4+ CD183+ CD45RO-
memory Th1 cells	CD3+ CD4+ CD183+ CD45RO+
naïve Th2 cells	CD3+ CD4+ CD45RO- CRTH2+
memory Th2 cells	CD3+ CD4+ CD45RO+ CRTH2+
regulatory T cells	CD3+ CD4+ CD25+ CD127-
**NK cells**	
natural killer cells	Lin- CD335+ CD56+ CD16+
memory-like natural killer cells	Lin- CD335+ CD56^dim^ CD16+
intermediate natural killer cells	Lin- CD335+ CD56^bright^ CD16-
innate natural killer cells	Lin- CD335+ CD56- CD16-

#### 2.7.3 Cytokine Profiles

Plasma cytokine profiles will be measured by Luminex^®^ xMAP™ technology (Austin Texas, USA) since they reflect the activity of various immune cells (**Table 7**). Luminex^®^ xMAP™ technology performed on MAGPIX™ instruments, enables the simultaneous quantification of up to 50 target proteins or nucleic acids (80) and have been validated for numerous immunological studies, including breast cancer (20, 62, 81) and infections (82). Superparamagnetic microsphere beads are conjugated with a distinct monoclonal antibody. The beads themselves are dyed with varying amounts of red and infrared fluorophores to allow clear assignment to a specific bead region. MAGPIX™ fluorescence imager-based instruments use a LED illumination/CCD camera detection system for both bead region identification and reporter fluorophore-based analyte quantification (83).

**Table 7:** Cytokine profiles measures by Luminex™ (MAGPIX^®^).

Cytokine	abbreviation
Tumor necrosis factor α	TNFα
Interferon γ	IFNγ
Interleukin 1α	IL-1α
Interleukin 1β	IL-1β
Interleukin 2	IL-2
Interleukin 4	IL-4
Interleukin 6	IL-6
Interleukin 10	IL-10
Interferon γ -induced protein 10	IP10
Monocyte chemoattractant protein 1	MCP-1
Granulocyte-macrophage colony-stimulating factor	GM-CSF

#### 2.7.4 Gene Expression Profiles

The expression of selected mRNA transcripts related to breast cancer and inflammation will be measured by a quantitative real-time PCR (qPCR). The method used is based on a previously published technique (73). Total RNA will be isolated from PMBCs using the High Pure RNA Isolation Kit (Roche, Basel, Suisse). Reverse transcription will be performed using SuperScript™ VILO IV cDNA Synthesis Kit (Invitrogen, Thermo Fisher Scientific) followed by the cDNA purification using QIAquick™ PCR Purification Kit (Qiagen, Hilden). Concentration and purity determination of the isolated RNA will be performed with NanoDrop™ (Thermo Fisher Scientific, Waltham, Massachusetts, USA). TaqMan™ Gene Expression Assays (Applied Biosystems, Thermo Fisher Scientific, Waltham, Massachusetts, USA) will be used to perform qRT-PCR of immunological key transcription factors TBX21, RORC, GATA3, FOXP3 (84–88). The Δct-values will be calculated using ACTB, EEF1A1, and 18S as a standard. Values will be normalized to the data from the samples taken at the baseline visit.

### 2.8 Assessment of Psychological Parameters

The assessment of psychological parameters will be performed by using standardized questionnaires that were validated for (breast) cancer patients, respectively (89–98). A list of questionnaires is shown in **Table 8**. Moreover, the patients have the opportunity to report individual thoughts as free texts and during the study visits.

**Table 8:** Assessment of the psychological parameters (used questionnaires).

Abbreviation	Name	Nmber of items	Content	Reference
**EORTC QLQ-C30**	European Organization for Research and Treatment of Cancer Quality of Life Questionnaire-Core 30	30 items	health-related quality of life (QoL)	(95)
**EORTC QLQ-BR23**	European Organization for Research and Treatment of Cancer Quality of Life Questionnaire-BReast cancer module 23	23 items	health-related quality of life (QoL) in breast cancer patients: systemic therapy side-effects, arm symptoms, breast symptoms, body image and sexual functioning	(95)
**MHS**	Mental Health Scales	76 items	willpower, acceptance of life, self-reflection, finding of a meaning, naturalness and social integration	(92)
**HADS**	Hospital Anxiety and Depression Scale	14 items	level of anxiety and level of depression	(89)
**DT**	Distress Thermometer	DT: 1 item	Psychosocial distress and possibly associated problems (practical, family, emotional, spiritual/religious and physical)	(94)
		Problem list: 39 items		
**MDWQ**	MultiDimensional Well-being Questionnaire	12 items	mood, level of alertness and level of calmness	(91)

#### 2.8.1 Quality of Life

The overall quality of life is assessed using the Quality of Life Questionnaire (QLQ-C30 version 3.0) which has been developed and validated by the European Organisation for Research and Treatment of Cancer (EORTC) to explore quality of life among cancer patients (90). The supplementary questionnaire QLQ-BR23 will be used to record symptoms specific for breast cancer (95). In sum, a score from 0 – 100 can be calculated allowing the quantification of the impairments regarding the patient’s health and quality of life.

#### 2.8.2 Mental Health

The questionnaire “Mental health scales” (MHS) (92) was used to assess the psychological integrity of the study participants, in order to evaluate the development of the patients’ personality throughout the study duration. The questionnaire consists out of seven scales (autonomy (17 items), willpower (14 items), acceptance of live (8 items), self-reflection (12 items), finding of a meaning (7 items), naturalness (10 items) and social integration (8 items)), which yields a total of 76 items. The questionnaire is answered with the help of a five-point Likert-scale (*1* = *I fully agree*; *5* = *I fully disagree*) and an example item is “Generally I am confident”.

#### 2.8.3 Chronic Fatigue Syndrome

Symptoms of chronic fatigue syndrome (CFS) (93) that affects more than 50% of breast cancer patients and the extent of psychological stress will be assessed using the Distress Thermometer (DT) (96).

#### 2.8.4 Anxiety and Depression

The self reported anxiety and distress will be assessed using the HADS (Hospital Anxiety and Depression Scale) questionnaire (89, 97) and the German adaptation of the Distress Thermometer (DT) (94, 96–98).

#### 2.8.5 Well-Being

To get an overview of the mental state of the patients, the German questionnaire “Multidimensional Well-being Questionnaire” (MDBQ) (91) has been used in its short version with twelve items (short version A). This questionnaire captures three bipolar dimensions of the current mental state (good mood – bad mood, alertness – fatigue, tranquility – inquietude) of the breast cancer patients. The questionnaire presents twelve adjectives (e.g., satisfied, flabby, good etc.) and with the help of a five-point Likert-scale (*1* = *not at all*; *5* = *very much*) the patients rate their instant feeling. Cronbachs’α is between 0.86 and 0.94, indicating a good consistency of the scale.

### 2.9 Sample Size Calculation and Statistical Analyses

The Institute for Medical Biometry, Epidemiology and Medical Informatics (IMBEI) Saarland University is supporting the sample size calculation, study design, data management and evaluation using PASS 2019 (NCSS, LLC, Kaysville, Utah, USA) and SPSS (Version 27 IBM SPSS Statistics, Armonk, New York, USA). Data were collected by using Excel 2019 (Microsoft, Redmond, USA). A sample size of 110 produces a two-sided 95% confidence interval with a distance from the mean to the limits that is equal to 2.835 when the estimated standard deviation is 15.0. A sample size of 110 produces a two-sided 95% confidence interval with a width equal to 0.187 when the sample proportion is 0.50.

Continuous measures are presented as means ± standard deviations (SD) or medians (range). Categorical variables are presented as frequencies (percentage). For continuous variables normality is tested using Shapiro-Wilk-Test. In case of non-rejection of normality two-group comparisons are due to the t-test for two independent samples. For more than two groups, comparisons are due to one-way Analysis of Variance (ANOVA). Comparing two repeated measurements, t-test for 2 dependent samples is used and for more than two we use repeated measures ANOVA. In case of rejection of normality, Mann-Whitney U-test, Kruskal-Wallis-test, Wilcoxon test for two dependent samples and Friedman-test are used, respectively. For group comparisons considering categorical variables the chi-squared test and for repeated measures McNemars’s test are applied. Considering possible confounding we use subgroup analyses for categorical subgroups or propensity-score-matching otherwise. Statistical significance will be calculated using appropriate statistical methods with two-sided p-values < 0.05. The statistical analyses are explorative, so there will be no correction for multiple testing.

Data cleanup will include tests for missing data and plausibility. Depending on the degree of incompleteness or lack of accuracy, all, or some of the data from a patient will be excluded from further evaluation. In the case of a study drop out prior the last follow up visit, the patient’s data will only be included, and tests will be performed to identify potential predictors for a study drop-out (e.g., age or weight) that might introduce a bias. The risk of bias will be discussed in any publications of the data.

## 3 Discussion

The BEGYN study will provide a holistic insight into the physical activity in relation to the physiological and psychological dynamics during the first year after diagnosis of non-metastatic breast cancer. The complex interplay between the underlying disease, antineoplastic therapy and individual constitution affects the patient *in toto*. Thus, therapeutic approaches must not be limited to surgery, antineoplastic medication, radiotherapy and psychological intervention alone, but should include the motivation for supportive activities such as exercise, which has great effect on the quality of life and prognosis (35, 99–101). A better knowledge of biomarkers is a prerequisite to assess the effects of complementary therapies and to develop personalized strategies for rehabilitation, e.g., regarding the type and dose of exercise (9). Therefore, the BEGYN study unites multiple validated assessments, allowing cross-linking analyses between the level of physical activity, cardiopulmonary fitness, body composition, biochemical measurements, an in-depth immune status including quantification of circulating lymphocyte subpopulations, mRNA expression and cytokine profiles and an extensive evaluation of psychological factors.

### 3.1 Novelty of the Approach

Whereas most studies investigating the influence of physical activity in breast cancer patients focus on specific activities for three months or less, the BEGYN study will quantify the daily physical activity and cardiorespiratory fitness of breast cancer patients based on objective measurements in the context of the oncological therapy for 12 months after diagnosis. Multiple studies have shown that physical activity can have positive effects in breast cancer patients (4–6, 33–35, 37). Guidelines recommend physical activity during breast cancer treatment, in example in the United States (102), in Great Britain (103), in Germany (39) and others. In particular, physical activity appears to have a positive effect on breast cancer associated fatigue syndrome (3) and on the overall quality of life (104). However, multiple aspects including the underlying mechanisms remain poorly understood. The BEGYN study will contribute important data to understand the influence of physical activity on the dynamics during the first year of breast cancer treatment and may reveal potential modifiers of the patient’s well-being.

#### 3.1.1 Holistic Approach

To our knowledge, the BEGYN study is one of the largest studies with a very broad approach in breast cancer patients that will allow to identify associations between physical, psychological and laboratory variables, thus linking aspects that are often studied separately in breast cancer patients. Regarding the assessment of physical activity, it will be of particular interest to discuss the results of the BEGYN study in the light of other ongoing highly innovative studies such as the PROTECT study which also includes patients with colorectal cancer and lung cancer (6). One of the major strengths is the continuous recording of the patient’s physical activity and well-being by diary and by use of a fitness tracker since physical activity encompasses not only explicit sports activities, which may last a few hours per week, but also activity during everyday activities. Various forms of physical activity have been proposed, such as yoga, Tai Chi Chuan, Nordic walking, jogging, weight training, cycling, dancing and many others (20, 36, 37, 47). However, it has been claimed that individualized recommendations are needed to meet the needs of the patient (6, 105). By using the concept of metabolic equivalents (MET), the BEGYN study will allow comparing effects of physical activities of similar intensities independent of the type of exercise. Since nutrition, food supplements and lifestyle may significantly influence the effects of exercise and antineoplastic therapies, these variables are assessed by using questionnaires and the self-assessment diary.

#### 3.1.2 One Year Study Schedule

The BEGYN study will yield an overview on a relatively long period of 12 months after diagnosis, starting before any specific antineoplastic therapy. Thus, the patient will typically be observed beyond the initial steps of therapy, which are also often associated with psychoemotional distress. This may give insight into predictors of a more favorable long-term management towards a new physical and psychological balance. Moreover, the 12 months study period will reduce potential artifacts due to seasonal effects which can, in example, influence outdoor activities and Vitamin D concentrations. The role of Vitamin D in carcinogenesis and cancer therapy is still under debate. Importantly, serial measurements of 2,5 OH Vitamin D concentrations in peripheral blood and assessment of therapeutic Vitamin D intake may give insight into the role of Vitamin D metabolism and may also provide data on the interaction between antineoplastic therapy and Vitamin D. In addition, the BEGYN study will yield serial selenium concentrations and insight on the consumption of selenium by the patients, which often is provided as a self-medication, causing significant financial burden for some patients.

#### 3.1.3 Comparison of Different Antineoplastic Therapies

The BEGYN study will allow to compare patients with various antineoplastic therapies, i.e., endocrine therapy, chemotherapy, surgery, and radiotherapy. Due to the strict adherence to the national guideline, therapy concepts are to be expected representative for the German national standard.

#### 3.1.4 Immunophenotyping

The BEGYN study will yield a deep insight into the innate and adaptive immune system during the first year after diagnosis of non-metastatic breast cancer (62). Inflammatory processes are a crucial part of the physiological response to cancer cells. Simultaneously, the BEGYN study will shed light on the effects of endocrine therapy and of chemotherapy on the immune system. Moreover, it is well recognized that moderate physical activity is associated with an improved immune status whereas excessive physical activity leads to an increase susceptibility towards (viral) infections (22). Interestingly, natural killer (NK) cells play a key role in sports physiology and in restricting tumor growth (64). Thus, the BEGYN study will be among the first studies to quantify four distinct subsets of NK cells in relation not only to breast cancer and antineoplastic therapy, but also in relation to the extent and type of physical activity.

The BEGYN study also has some limitations, such as the single center approach. However, the data will serve as a basis for designing future multicenter studies. In example, the results of the BEGYN study might help focusing the highly detailed immunophenotyping regarding the characterization of leukocyte subsets, mRNA transcripts and cytokines to the most promising variables in future study protocols. One further limitation of the study is that the quality of self-assessments may underlie intra-individual and inter-individual variations when filling out the self-assessment diary (e.g. daily documentation of physical activities, weekly read outs of the measurements of the fitness tracker etc.). Due to the need of serial measurements of the body composition, ethical concerns on X-ray exposure were the reason to use bioimpedance analysis and plicometry rather than Dual-energy X-ray absorptiometry, which is regarded as the gold standard (106). With regard to the known limitations of bioimpedance analysis and plicometry, we will compare the intra-individual relative changes over time instead of absolute values (107). In addition, we will further extend the data by estimating the body composition from the routine CT scans of the study patients (56). The BEGYN study is an observational study, thus potential causal relationships must be interpreted carefully. Ethical concerns would not allow randomizing patients into a group that would not be encouraged to exercise since this would conflict with the high evidence guidelines. However, the BEGYN study might help defining valid methods of continuous registration of physical activity for future studies to compare the role of various types of exercise, e.g., training of strength versus endurance.

### 3.2 Conclusion

The holistic approach over 12 months including physiological, psychological, and immunological data is the main strength of the BEGYN study. By including a homogeneous group of 110 female non-metastatic breast cancer patients, the study will provide highly valid data on the complex interplay between physical activity, underlying disease, type of therapy and psychological parameters.

The BEGYN study provides a uniquely thorough analysis of non-metastatic breast cancer patient during the first year after diagnosis.

### 3.3 Ethics and Dissemination

The study is carried out at the Department for Gynecology, Saarland University Medical Center and has been approved by the ethics committee of the Medical Association of Saarland (study # 229/18). Written consent is obtained from the patient in accordance with the Declaration of Helsinki. Any amendment to the protocol will require the formal modification and approval by the same local ethics committee that approved the study prior to implementation and will be described transparently in subsequent reports. This study is registered at German Clinical Trials Register (DRKS) (DRKS00024829). Patient recruitment took place between September 2019 and January 2021 and data collection will continue until March 2022.

## Ethics Statement

The studies involving human participants were reviewed and approved by ethics committee of the Medical Association of Saarland (study # 229/18). The patients/participants provided their written informed consent to participate in this study.

## Author Contributions

CZ designed the study and wrote the first draft of the manuscript. GW performed sample size calculations, gave advice for statistical analyses and helped drafting the manuscript. CS, JS, CW, CM, LA, ML, L-SS, LK, and IT performed the clinical experiments, helped with the study design, and helped writing the manuscript. CM helps to raise funding and helped writing the manuscript. EK, RS, and SG-F performed the laboratory experiments and helped writing the manuscript. MZ, GS, and E-FS gave advice for the study design, supervised the study, and helped writing the manuscript. All authors contributed to the article and approved the submitted version.

## Funding

This work was supported by *miteinander gegen Krebs e.V.* and by the intramural funds of the Saarland University Medical Center.

## Conflict of Interest

The authors declare that the research was conducted in the absence of any commercial or financial relationships that could be construed as a potential conflict of interest.

## Publisher’s Note

All claims expressed in this article are solely those of the authors and do not necessarily represent those of their affiliated organizations, or those of the publisher, the editors and the reviewers. Any product that may be evaluated in this article, or claim that may be made by its manufacturer, is not guaranteed or endorsed by the publisher.
